# Effectiveness and experiences of the Extension for Community Healthcare Outcomes (ECHO) Model in developing competencies among healthcare professionals: a mixed methods systematic review protocol

**DOI:** 10.1186/s13643-021-01832-0

**Published:** 2021-12-16

**Authors:** Gabrielle Chicoine, José Côté, Jacinthe Pepin, Guillaume Fontaine, Marc-André Maheu-Cadotte, Quan Nha Hong, Geneviève Rouleau, Daniela Ziegler, Didier Jutras-Aswad

**Affiliations:** 1 Faculty of Nursing, Université de Montréal, Pavillon Marguerite-d’Youville, C.P. 6128 succ. Centre-ville, Montreal, QC H3C 3J7 Canada; 2 Université de Montréal Hospital Research Centre, Montreal, QC Canada; 3 Research Chair in Innovative Nursing Practices, Université de Montréal Hospital Research Centre, Montreal, QC Canada; 4 FUTUR Team-FRQSC, Faculty of Nursing, Université de Montréal, Montreal, QC Canada; 5 Centre for Implementation Research, Psychology and Health Research Group, Clinical Epidemiology Program, Ottawa Hospital Research Institute, Ottawa, ON Canada; 6 Research Centre, Montreal Heart Institute, Montreal, QC Canada; 7 EPPI-Centre, UCL Social Research Institute, University College London, London, England; 8 Women’s College Hospital, Toronto, ON Canada; 9 Faculty of Medicine, Department of Psychiatry and Addiction, Université de Montréal, Montreal, QC Canada

**Keywords:** Project ECHO, Telementoring, Videoconferencing, Continuing medical education, Distance learning, Virtual collaboration, Virtual community, Knowledge-sharing community

## Abstract

**Background:**

The Extension for Community Healthcare Outcomes (ECHO) Model of continuing tele-education is an innovative guided-practice model aiming at amplifying healthcare professionals’ competencies in the management of chronic and complex health conditions. While data on the impact of the ECHO model is increasingly available in the literature, what influences the model effectiveness remains unclear. Therefore, the overarching aim of this systematic review is to identify, appraise, and synthesize the available quantitative (QUAN) and qualitative (QUAL) evidence regarding the ECHO Model effectiveness and the experiences/views of ECHO’s participants about what influences the development of competencies in healthcare professionals.

**Methods:**

The proposed systematic review was inspired by the Joanna Briggs Institute (JBI) methodology for Mixed Methods Systematic Reviews (MMSR) and will follow a convergent segregated approach. A systematic search will be undertaken using QUAN, QUAL and mixed methods (MM) studies of ECHO-affiliated programs identified in six databases. A publication date filter will be applied to find the articles published from 2003 onwards. Sources of unpublished studies and gray literature will be searched as well. Retrieved citations will independently be screened by two reviewers. Disagreements will be resolved through discussion until a consensus is reached or by including a third reviewer. Studies meeting the predefined inclusion criteria will be assessed on methodological quality and the data will be extracted using standardized data extraction forms. Separate QUAN and QUAL synthesis will be performed, and findings will be integrated using a matrix approach for the purpose of comparison and complementarity.

**Discussion:**

This MMSR will fulfill important gaps in the current literature on the ECHO Model as the first to provide estimates on its effectiveness and consider simultaneously the experiences/views of ECHO’s participants. As each replication of the ECHO Model greatly varies depending on the context, topic, and targeted professionals, a better understanding of what influences the model effectiveness in developing healthcare professionals’ competencies is crucial to inform future implementation.

**Systematic review registration:**

PROSPERO CRD42020197579

**Supplementary Information:**

The online version contains supplementary material available at 10.1186/s13643-021-01832-0.

## Background

### Innovation in continuing education: the ECHO model

In the current context of change and uncertainty, healthcare professionals are expected to develop high levels of competencies to effectively manage complex health conditions and respond to populations’ multiple needs [1]. Further, healthcare professionals’ competencies such as leadership, clinical reasoning, ethical attributes and effective teamwork are founded at the premise of safety, quality, and accessibility improvements in healthcare [2, 3]. In the health education science literature, the definition of competency remains polysemous due to varying conceptions and underlying philosophical assumptions [4]. However, authors mostly agree that a competency: (1) requires the efficient mobilization and orchestration of a cluster of internal resources (e.g., knowledge, attitudes, values, skills, abilities) and external resources (e.g., material, human, organizational) in clinical practice; (2) is constantly contextualized to a specific situation; and (3) evolves throughout a professional’s lifetime [5–10]. Hence, a competency can be understood as complex and systemic knowledge in action to effectively solve real-life situations [11, 12].

Situated within clinical practice, an example of the competency “performing a holistic evaluation of a patient's needs” may require for healthcare professionals to efficiently mobilize different internal and external resources. Internal resources include anterior knowledge on health conditions, communication skills, non-judgmental attitudes and critical thinking to detect risk and complications, while external resources involve clinical tools, evidence-based guidelines, and support from colleagues.

Competency development refers to a dynamic and ongoing process of learning and practice renewal requiring engagement at individual and collective levels [7, 13, 14]. It is crucial in ensuring healthcare professionals practice within the full scope of their role and in improving patients’ health outcomes [2, 3]. In the healthcare professions, continuing education (CE) is recognized as an essential aspect of competency development [15–17]. CE can be described on a continuum, from informal learning experiences and practices to formal educational interventions held through a diversity of modalities and sources of media, in both academic and clinical practice settings [18]. In all cases, CE focuses on meaningful learning experiences that are conducive to competency development in healthcare professionals.

In recent decades, several continuing educational programs using information and communication technologies (ICTs) have been developed to overcome barriers related to healthcare professionals’ participation in CE activities (e.g., staff shortages, cost, travel time) [19–25]. Advantages of ICT-based programs include increased accessibility, lower costs and personalization compared to large-group, in-person instruction [26]. One of these ICT-based programs is the *Extension for Community Healthcare Outcomes* (ECHO) Model [27], a continuing tele-education program that provides ongoing support and clinical supervision to healthcare professionals in the management of complex and chronic health conditions.

### Description and conceptual representation of the ECHO Model

Launched in 2003 at the University of New Mexico Health Center, ECHO aims to facilitate knowledge sharing and capacity building, and expand access to best practice care to reduce treatment disparities in underserved populations. The ECHO Model was first developed under the name of Project ECHO (© 2020, The University of New Mexico, Albuquerque, NM, USA; http://www.hsc.unm.edu/echo) to support primary care providers in rural and carceral settings in managing patients infected by the Hepatitis C virus [28–33]. Since then, the model has been replicated for dozens of diseases and health conditions and now operates at more than 100 academic medical health centers across multiple continents [9]. The ECHO Model involves establishing a network between front-line healthcare professionals located in remote areas—i.e., “spokes”—with a multidisciplinary team of specialists at academic medical centers—i.e., a “hub”—using videoconference technology. The model typically includes a 6-to-12-month curriculum of weekly “ECHO clinics”, in which a case-based discussion about a real patient situation and a short didactic presentation are held over 2 h.

**Fig. 1:**
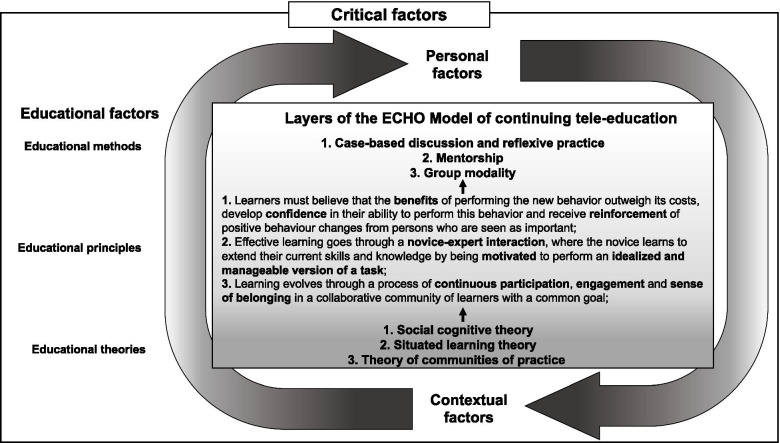
Layers of Cianciolo and Regehr’s Framework [34] applied to the ECHO Model

To offer a meaningful understanding of the ECHO Model, we developed a conceptual representation that explores the intended function of the model in context—meaning an examination of surface-level components with the model’s conceptual learning conditions. This conception builds on Cianciolo and Regehr’s *Learning Theory and Educational Intervention Framework* [34], a layered perspective based on the premise of enabling a rich examination of the interplay between the pedagogic intention of an educational intervention (i.e., educational theories and principles) and its adaptation in a specific context (i.e., educational methods, personal and contextual factors). The authors claim that this examination helps to discern whether a given educational intervention “worked” as intended on an anticipated outcome and to draw any plausible conclusions about which components of the intervention this effect can be attributed to. Figure 1 depicts the proposed layered conceptualization of the ECHO Model that is summarized in the paragraphs below.

As shown in Fig. 1, our conception excavates the layers of the ECHO Model: methods at the surface, principles in the middle, and theories at the core. Together, these three layers depict the favorable educational conditions that must be established in a program for learning to occur. This layered classification illustrates that the ECHO Model has a unique identity, which preserves its essence despite being adapted in a specific context. Also, the lack of clear delineations between layers reflects the absence of clear boundaries between them.

At the bottom of Fig. 1, the layer of educational theories indicates that the ECHO Model is based on three educational theories, which together constitute the foundational layer or identity of the model: (1) Social Cognitive Theory [35]; (2) Situated Learning Theory [36, 37]; and (3) Theory of Communities of Practice [38]. According to Cianciolo and Regehr [34], this foundational theory layer represents a context-independent and idealized statement of the educational conditions that must hold for a given intervention to be what its designers claim it is—i.e., the pedagogic intention.

The middle layer of educational principles illuminates the general underlying postulates that are engaged throughout an intervention and clarifies the structural learning aspects of the ECHO Model—i.e., assumptions of how participants will learn [39]. These principles may be adjusted to context, but nevertheless they reflect relatively stable approaches to learning. The top layer of the figure, the most context-sensitive, comprises educational methods that account for context, allowing each ECHO model replication to be tailored to a specific local setting (e.g., modes of delivery, educational strategies, schedule, functioning). The educational methods used in the ECHO Model are summarized in three main categories, as shown in Fig. 1. Each category specifies the type of learning experience a participant might be exposed to—meaning the intervention components/characteristics. Concrete examples of how these educational methods are delivered in ECHO implemented programs [28] can be found in Additional file 1: Table 1.

Our conceptualization also suggests that implementation comprises a complex and uncontrolled set of critical factors that influence the adaptation of a given educational program in a specific context. Hence, the arrows surrounding Fig. 1 imply that personal and contextual factors, as an overlay to the maintenance of the conceptual educational conditions of the ECHO model, may influence its effectiveness, sometimes in unanticipated ways. Importantly, our conception reflects the fact that personal and contextual factors are seen as processes of influence on learning. Specifically, personal factors refer to influences from an individual perspective, while contextual factors range from the proximal influences (e.g., interpersonal) to increasingly distal influences (e.g., institutional, organizational, community, and sociopolitical) [40, 41]. In summary, the proposed conception supports the contention that a deeper investigation of the ECHO Model’s effectiveness must be undertaken within a holistic and rich examination of what influences the development of competencies.

### Current knowledge gaps about the ECHO Model and importance of the proposed mixed methods systematic review

There is substantial evidence showing the positive impacts of the ECHO Model on healthcare professionals’ learning outcomes [9, 42]. For instance, increases in healthcare professionals’ perceived knowledge and confidence in their ability to manage complex cases without referring to specialists as well as improvements in their ability to perform new behaviors in practice have been reported [9, 42]. Moreover, there is evidence in support of the acceptability and feasibility of the model, notably for reducing healthcare professionals’ feelings of isolation and regarding its cost-effectiveness [42]. The relevance of the didactic presentations’ topics for practice, peer-to-peer interactions and positive reinforcement from specialists were reported as favorable conditions in amplifying healthcare professionals’ knowledge and skills [9, 43]. Additionally, mentorship led by a qualified and competent team of healthcare professionals—the “hub”—in a helping environment has been found to support ECHO’s participants in difficult clinical situations and allow them to reflect on their own practice [43].

A preliminary search of MEDLINE, PROSPERO, the Cochrane Database of Systematic Reviews and the *Joanna Briggs Institute (JBI) Database of Systematic Reviews and Implementation Reports* was conducted to identify potentially relevant reviews and empirical studies. Only two systematic reviews focusing on the impact of the ECHO Model on healthcare professionals’ learning outcomes and patients’ health outcomes were retrieved [9, 42].

In the first review (*N* = 39), Zhou et al. [42] synthesized the data of ECHO-affiliated programs from published and unpublished studies between 2003 and 2015. The authors concluded that the model positively impacted healthcare professionals, exclusively for the parameters of participation, satisfaction, knowledge and self-efficacy. In addition, the review compiled data from two qualitative (QUAL) studies and found that increasing one’s knowledge base, applying new knowledge to future patients and collaborating with specialists were motivating factors to participating in ECHO, while the main barriers reported were lack of time and videoconference technology. However, given that this review included a majority of quantitative (QUAN) observational studies such as single group cohort studies and pre-post design studies, the effectiveness of ECHO on healthcare professionals and patients’ outcomes is less clear.

In the second systematic review (*N* = 52), McBain et al. [9] aimed to gather evidence published between 2000 and 2018 on a broad spectrum of ECHO and “ECHO-like” models. The authors found that the ECHO Model and similar adaptations favorably impacted healthcare professionals’ satisfaction, knowledg; and confidence. Concerning patient-related outcomes, 11 studies incorporated a comparison group and none involved randomization. The authors concluded on a general effect at improving outcomes in the case of patients with hepatitis C, chronic pain, dementia, and type 2 diabetes. However, the inclusion of non-affiliated ECHO programs produced heterogeneity between studies that precluded further statistical analyses.

Although both reviews have the strength of providing a knowledge synthesis from different types of empirical studies, little attention was paid on the integration of QUAN and QUAL evidence. In other words, the insight or added value gained from combining/comparing/contrasting the resultant QUAN and QUAL findings of the review is unclear. Without the integration of QUAN and QUAL results, a complete picture of the complexities associated with the ECHO Model effectiveness on healthcare professionals’ competencies is currently lacking [44]. It must also be recognized that since the completion of both reviews, our preliminary search of the databases retrieved three randomized controlled trials [RCTs] and 10 non-randomized controlled studies of ECHO programs, as well as 24 QUAL and mixed methods [MM] studies in which the experiences/views of ECHO’s participants were explored. In addition, we have identified five other QUAL and MM studies published between 2012 and 2018 that were not included in both reviews. The evidence from all those studies have not been systematically reviewed and synthesized.

Considering that ECHO-affiliated programs have been implemented in diverse settings—each implementation bringing variations to the original Project ECHO—and that the model is intended for a wide range of clinical conditions and professional groups, our current understanding of what has contributed to the observed studies’ outcomes is limited. In order to address these knowledge gaps, it has been suggested that there is a need to not only assess the ECHO Model effectiveness from QUAN randomized and non-randomized controlled studies, but also to examine what influences the model success (or failure) [45]. To our knowledge, no previous or in-progress review has focused on investigating what influences the ECHO model effectiveness in developing healthcare professionals’ competencies. Therefore, a mixed methods systematic review (MMSR) is needed to combine the findings of QUAN studies on the ECHO Model effectiveness together with QUAL evidence on the experiences/views of ECHO’s participants. This will allow a better understanding of whether and under what influences ECHO works (or not).

### Aim and review questions

The aim of this MMSR is to identify, appraise and synthesize the available QUAN and QUAL evidence regarding the effectiveness and experiences of the ECHO Model in developing competencies among healthcare professionals. This systematic review seeks to answer the following three questions:

- What is the effectiveness of the ECHO Model on healthcare professionals and patients’ outcomes? (QUAN question)
- What are the experiences/views of ECHO’s participants, including both mentees and mentors, about what influences the development of competencies in healthcare professionals? (QUAL question)
- What can be inferred from the QUAL synthesis on the experiences/views of ECHO’s participants that can explain the ECHO Model effectiveness in developing healthcare professionals’ competencies? (Mixed methods [MM] question)

## Methods

This systematic review protocol was inspired by the JBI methodology for MMSR [46] and is reported according to the Preferred Reporting Items for Systematic Reviews and Meta-Analysis Protocols (PRISMA-P) 2015 checklist [47, 48] together with the PRISMA 2020 updated guidance [49] (see Additional file 2).

### Approach

**Fig. 2:**
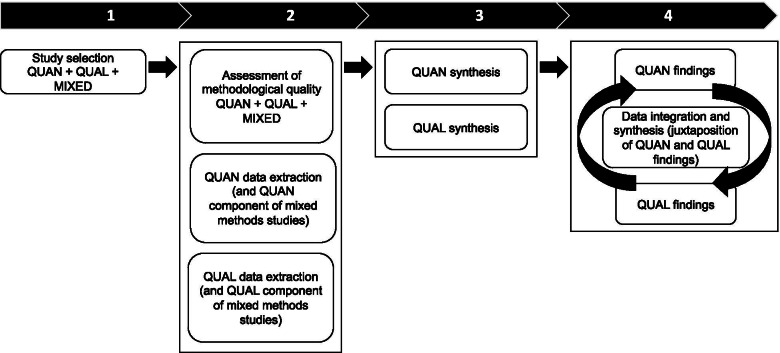
The convergent segregated approach to MMSR inspired by Lizarondo et al. [46] and Pluye et al. [51]

We will use a convergent segregated approach [46, 50] to extract and synthesize data from the QUAN, QUAL and MM included studies (see Fig. 2). With this type of design, the QUAN and the QUAL extracted data will be first analyzed separately using different synthesis methods (QUAN descriptive statistics and synthesis, intervention effect estimates, and QUAL thematic synthesis) and then the findings of both syntheses will be merged for the purposes of comparison and complementarity [51].

### Eligibility criteria

In accordance with the PICO (Participants, Intervention, Comparator, Outcomes) mnemonic for the QUAN component and the PICo (Population, phenomenon of Interest, Context) mnemonic for the QUAL component [46], studies will be screened based on the following criteria:

#### Participants

We will include QUAN studies (or QUAN component of MM studies) conducted with healthcare professionals participating in ECHO as mentees, including registered and allied health healthcare professionals (e.g., community health providers, long-term care providers, pharmacists, physicians, psychologists, nurse practitioners, nutritionists, physiotherapists, social workers, and occupational therapists), and regardless of their practice area (e.g., family medicine, geriatric care, addiction and psychiatric services, pain management, and pediatric care). Given that the ECHO program continuously operates and that participation in each videoconferencing session for an entire curriculum is not required, studies in which mentees did not complete the program will be included in this review. We will exclude studies that involve pre-licensure healthcare professional students only, since entry-to-practice competencies may differ from the standards and levels of competencies in clinical practice.

#### Intervention

For inclusion in the QUAN component of this review, we will consider studies of ECHO-affiliated programs targeting healthcare professionals and in which the effectiveness of the program was assessed. We defined ECHO programs as “a technology-enabled collaborative learning and capacity building model” [52]. This type of distance health education model connects specialists (i.e., the mentors) with multiple other healthcare professionals (i.e., the mentees) through simultaneous interactive videoconferences for the purpose of strengthening their knowledge and competencies in providing high-quality healthcare. In accordance with this definition, the following six inclusion criteria will be used for considering programs as “ECHO-affiliated”: (1) using a technology-enabled platform (videoconferencing sessions); (2) having a health-focused objective; (3) implementing a hub-and-spoke framework with generalists in one or more locations (spokes) and specialists at a different location (hub) with a ratio of more than 1:1; (4) using case-based learning (presentation and discussion of a real patient case); (5) using interactive mentorship; and (6) including a didactic component. Technology-enabled collaborative learning and capacity building models that are not “ECHO-affiliated” will be excluded. We will include studies of implemented ECHO programs addressing any type of health conditions and topics (e.g., chronic pain, co-occurring disorders of mental health and substance use disorders, delirium, diabetes, infection diseases).

#### Comparator

We will consider for inclusion QUAN studies (or QUAN component of MM studies) with all types of comparator(s), including both active and passive comparison groups. An active comparison group will be defined as a set of participants who receive one or more interventions planned as part of their participation in research, and not received by participants in the ECHO intervention group. A passive comparison group will be defined as a set of participants in whom no specific intervention is offered as part of their participation in the research (or any intervention that is not received by participants in the ECHO intervention group).

#### Outcomes

The New World Kirkpatrick Model [53] is an evaluation model for educational programs that is frequently used in the healthcare professions. It was chosen to operationalize and categorize the outcomes for this systematic review. The model includes the following four levels of outcomes: (1) reaction—i.e., the degree to which participants find the educational program favorable, engaging, and relevant to their practice; (2) knowledge—i.e., the degree to which participants acquire the intended knowledge based on their participation in the educational program; (3) behavior—i.e., the degree to which participants apply what they learned in the educational program in their practice; and (4) results—i.e., the degree to which targeted outcomes occur as a result of the educational program.

Based on this categorization and the focus given to the development of competencies in healthcare professionals, the QUAN component of this review will consider studies that include at least one of the following critical or important [54] outcome measures:

##### Critical outcome

- *Competencies*: Studies assessing changes in healthcare professionals’ competencies based on their participation in ECHO will be included in the QUAN component of this review. In the context of CE assessment, Moore and colleagues [55] have operationalized the concept of competence as “the degree to which participants show how to do what the CE activity intended them to be able to do” (p.3), which can be assessed using objective or subjective outcome measures. According to this definition, we will be including in this review both studies that report on the development of formal competencies based on their use in clinical or educational settings (i.e., simulation, observation), and studies reporting on participants’ self-reported competencies. We will consider for inclusion the following proxy outcome measurements for self-reported competencies: perceived confidence, self-efficacy, self-reported abilities, and intention to change [55].

##### Important outcomes

- *Reaction* (Kirkpatrick’s level 1): Healthcare professionals’ views and reactions to ECHO including outcome measures of satisfaction and participation (e.g., number of online participants, degree of retention, and attrition rate).
- *Knowledge* (Kirkpatrick’s level 2): Healthcare professionals’ knowledge acquisitions based on their participation in ECHO, including objective (i.e., knowledge test) and subjective (i.e., self-reported knowledge) outcome measures of knowledge.
- *Behavior* (Kirkpatrick’s level 3): Application of healthcare professionals’ new knowledge acquisitions in clinical practice based on their participation in ECHO, including objective outcome measures (e.g., interventions or tool utilization, initiation of treatment, patient charts, care performance indicators from administrative database) or subjective outcome measures (e.g., perceived clinical performance, self-reported change in care plan) of clinical performance.
- *Results* (Kirkpatrick’s level 4): Changes in patients’ health due to changes in the practice behavior of healthcare professionals participating in ECHO, including objective outcome measures (e.g., measures recorded in patient charts or administrative databases and access to care) or subjective outcome measures (e.g., measures from patient self-reports) of health indicators. The health indicators targeted for this review will include outcomes such as health behaviors, health status, and well-being, including physical and psychological health, social functioning, and treatment outcomes.

#### Population

For the QUAL component of this review, we will consider for inclusion studies on the experiences/views of any healthcare professionals participating in ECHO, including both mentees and mentors. We will include studies on any type of registered or allied health profession, and participants with all levels of work experience and educational background.

#### Phenomenon of interest

We will consider for inclusion in the QUAL component of this review studies in which the experiences/views of healthcare professionals participating in ECHO are investigated. In specific, we will include QUAL and MM studies (QUAL component only) that explore the experiences/views of ECHO’s participants, including both mentees and mentors, about what influences—positively or negatively—the development of competencies in healthcare professionals.

#### Context

For inclusion in this review (QUAN and QUAL components), we will consider studies conducted in any type of clinical setting (e.g., ambulatory clinics, community health centers, hospitals, long-term care facilities, primary care services), geographic location (e.g., rural or remote areas, urban located services, and carceral healthcare), or country.

#### Type of studies

This review will consider QUAN, QUAL and MM studies. Regarding QUAN studies, we will include both experimental studies (RCTs, cluster RCTs, crossover RCTs) and quasi-experimental studies (e.g., non-randomized controlled trials, cluster non-randomized controlled trials, cohort study with control group). QUAL studies will include designs such as phenomenology, grounded theory, ethnography, narrative inquiry, interpretative description, exploratory, and action research. All types of QUAL data sources will be included in this review (e.g., individual semi-structured interviews, observations, field notes, focus groups). MM studies will be considered for inclusion if the QUAN, QUAL, or both components meet the inclusion criteria mentioned above. All types of MM designs will be included (e.g., convergent, sequential exploratory, sequential explanatory).

This review will be limited to empirical studies in peer-reviewed journals as well as in the gray literature. Given the review team members’ language expertise and available resources, only full-text papers of English or French-language studies will be included. Case reports, study protocols, discussion papers, editorials and knowledge synthesis papers (e.g., MMSR, narrative reviews, rapid reviews, realist reviews, systematic reviews, scoping reviews) will be excluded. We will include studies published from 2003 onwards as the initial pilot-ECHO program was launched in 2003 [32].

### Search strategy for identification of studies

A systematic search strategy was developed in consultation with an experimented librarian (DZ) and was reviewed by a second librarian. The search strategy was built with the objective of locating studies on ECHO-affiliated programs exclusively. Therefore, the search combined specific words and expressions related to the ECHO Model (e.g., Extension for Community Healthcare Outcomes, ECHO, Project ECHO, SCAN-ECHO, TeleECHO). Given the absence of standardized indexing, we exploded the ECHO specific terminology with search term groups covering the following three domains: (1) healthcare professionals; (2) technology-enabled collaborative learning and capacity building model; and (3) hub-and-spoke model linking specialists with healthcare professionals. The ECHO-specific terminology and the search terms used for each domain were developed based on a previous systematic review on the impact of the ECHO Model [9].

A pilot search will be first executed in MEDLINE using the search terminology to check if the seminal papers on this topic will be captured in the search strategy. Following this pilot, the search strategy will be refined and then translated for each database using controlled vocabulary (MeSH, EMTREE, and others) and free-text searching. A publication date filter will be applied to find the articles published from 2003 onwards. Additional file 3 presents the complete search strategy. The following bibliographic databases will be searched:

- Cumulative Index to Nursing and Allied Health Literature (CINAHL COMPLETE), via EBSCO
- Evidence Based Medicine Reviews (All EBM Reviews), via OVID
- Excerpta Medical Database (EMBASE), via OVID
- MEDLINE, via OVID
- American Psychological Association PsycINFO (APA PsycINFO), via OVID
- Education Resources Information Center (ERIC, public access) (https://eric.ed.gov)

A forward citation tracking procedure—i.e., search articles that cited the included studies—will also be performed in Google Scholar. Sources of unpublished studies and gray literature will include ProQuest Dissertations and Theses and DART Europe E-theses Portal. For QUAN studies only, ClinicalTrials.gov and WHO International Clinical Trials Registry Platform will be searched via the Cochrane Library. Reference lists of included studies as well as existing reviews on the ECHO model [9, 42] will be manually scrutinized to identify any relevant studies for inclusion. Automated search updates will be set up in each database to ensure the inclusion of the latest publications in the field of the ECHO Model. All results of the search strategies will be transferred in an Endnote X9 file (© 2020 Clarivate Analytics, USA; www.endnote.com).

### Study selection, appraisal, and data extraction

A three-stage process including study selection, appraisal, and data extraction will be followed. Each stage of the process will be conducted by teams of two independent reviewers. Teams will be formed based on combining reviewers with complementary experience or expertise (e.g., QUAN research designs and QUAL research designs.

Throughout the study selection, quality appraisal, and data extraction stages, study authors will be contacted for additional information regarding eligibility criteria if necessary. Also, any disagreements arising between the reviewers will be resolved through discussion until consensus is reached. In the event of a persistent disagreement, a third reviewer will solve the conflict.

#### Study selection

Before the screening process, duplicates will be removed with EndNote X9 using a Bramer method for de-duplication of database search results for systematic reviews [56]. All identified records will be uploaded into the Covidence systematic review software (© 2019 Veritas Health Innovation Ltd, Australia; www.covidence.org). Reviewers will first independently screen titles and abstracts according to eligibility criteria. Then, full-text articles of all studies deemed eligible will be retrieved and assessed in detail against the eligibility criteria by two independent reviewers. The results of the search will be presented in a PRISMA flow diagram [49]. Excluded studies with reasons for exclusion will be reported in table form as well.

#### Methodological quality assessment

All included studies will be assessed by two independent reviewers for methodological quality using the Mixed Methods Appraisal Tool (MMAT) version 2018 (© 2018 MMAT, Canada; http://mixedmethodsappraisaltoolpublic.pbworks.com) [57–59].

The MMAT was specifically developed to assess the methodological quality of various study designs, including MM studies, and proposes a list of 25 criteria based on five categories of empirical studies. The interpretation of methodological quality will consist of a thorough analysis of the included studies, and an attribution of 20% for each of the five criteria established by the tool, totaling 100% in case of compliance with all criteria [60]. For this review purpose, included studies will be ranked as high (all or four out of five criteria met), moderate (three out of five criteria met) or low (two or fewer criteria met). All studies, regardless of their methodological quality will undergo data extraction and synthesis.

The results of critical appraisal will be reported in narrative form and will also be taken into consideration when discussing the final integrated review findings. According to the MMAT recommendations [54, 58, 61], the rating and rationale for each criterion of all included studies will be reported in table form.

#### Data extraction and management

Based on the JBI standardized QUAN [62] and QUAL [63] data extraction tools, two separate data extraction forms will be developed specifically for this systematic review. These data extraction forms will be iteratively validated by the entire team of reviewers to ensure their completeness and clarity. Before data extraction, the forms will be tested on a total of six randomly selected articles from the search strategy (two studies of QUAN method only, two studies of QUAL method only, and two studies of MM) and amended accordingly.

For the QUAN component, data will be extracted from QUAN and MM studies (QUAN component only) included in the review by two independent reviewers and will be managed with the Covidence systematic review software (© 2019 Veritas Health Innovation Ltd, Melbourne, Australia; www.covidence.org). Data extraction will include the following specific details:

- First and corresponding author(s) information, publication year, and country
- Study funding source(s)
- Study objective(s) and design
- Study population and health care setting
- Time of study, method(s) of data collection
- Planned and actual sample sizes
- Participation and response rate
- Results of significance to the QUAN review question (outcomes measures of competencies and levels 1 to 4 of Kirkpatrick’s model), including details on outcomes (definition, time points measured, missing data) and measurement (name of tool, measurement units, scales)

In addition, we will use the Guideline for Reporting Evidence-based practice Educational interventions and Teaching (GREET) 2016 checklist [64] to collect informal evidence regarding each ECHO programs included in this review [65]. The GREET checklist is comprised of 17 items which are recommended for reporting consistent information on educational interventions. For this review, the GREET checklist will serve as a data extraction template, thus enabling an in-depth examination of each ECHO program’s components/characteristics (e.g., health conditions or topics addressed in the program, learning objectives, duration and frequency of videoconference sessions, instructors’ degree, modes of delivery, materials, incentives) in the final review synthesis. Informal evidence about ECHO programs will be mainly captured from the method and/or discussion sections [65] of studies included in both the QUAN and QUAL component of the review.

For the QUAL data extraction, full texts of QUAL and MM studies (QUAL component only) included in the review will be uploaded into the MAXQDA Standard software version 2020.1 (© 1995-2020 MAXQDA, distribution by VERBI GmbH, Berlin, Germany; www.maxqda.com). MAXQDA is a convenient software to conduct QUAL data extraction and in-depth analysis from a variety of sources (e.g., PDF, text audio and video files, figures and images) [66].

The QUAL data extracted will include specific details about first and corresponding author(s), study funding source(s), publication year, and geographical location. We will also extract data regarding study aim and research question(s), population(s), context, philosophical or theoretical foundations, methodology and method(s) for data collection. Study results relevant for the QUAL question on experiences/views will be extracted for further analysis. The extracted results will include themes, categories, verbatim extracts, and/or illustrations. Data extraction of QUAL studies and MM studies (QUAL component only) included in the review will be performed by two reviewers, with each of them subject to repeated independent readings.

### Synthesis and integration of QUAN and QUAL findings

In accordance with the JBI convergent segregated approach to MMSR [46], a fourth-step procedure will be performed at the synthesis and integration stage. This procedure will involve separate QUAN and QUAL synthesis followed by integration of the QUAN findings and QUAL findings. Table 1 summarizes this procedure and is detailed below.

**Table 1:** Planned procedures in a convergent segregated approach, adapted from Pluye et al. [51]

Review question	Input	Technique	Output
**What is the effectiveness of the ECHO Model on healthcare professionals and patients’ outcomes? (QUAN question)**	**Informal evidence:** ECHO programs’ components/characteristics (from QUAN studies and QUAN component of MM studies) **QUAN results:** ***Critical outcome:*** Competency development ***Important outcomes:*** Corresponding to Kirkpatrick’s four levels of program evaluation (reaction, knowledge, behavior, and results)	Descriptive synthesis and meta-analysis (QUAN synthesis)	- Description of ECHO programs’ components/characteristics - Effect size measures of ECHO programs on critical and important outcomes
**What are the experiences/views of ECHO’s participants, including both mentees and mentors, about what influences the development of competencies in healthcare professionals? (QUAL question)**	**Informal evidence:** ECHO programs’ components/characteristics (from QUAL studies and QUAL component of MM studies) **QUAL results:** Experiences/views of ECHO’s participants about what influences the development of their competencies (from QUAL studies and QUAL component of MM studies)	Descriptive and thematic synthesis (QUAL synthesis)	- Description of ECHO programs’ components/characteristics - Themes relating to what influences the development of competencies in healthcare professionals participating in ECHO
**What can be inferred from the QUAL synthesis on the experiences/views of ECHO’s participants that can explain the ECHO Model effectiveness in developing healthcare professionals’ competencies? (MM question)**	Resultant QUAN and QUAL findings (from QUAN, QUAL and MM studies)	Juxtaposition of findings using a matrix approach (MM synthesis) for the purpose of exploring the correspondence between the ECHO Model effectiveness (QUAN synthesis) and the experiences/views about what influences the development of competencies in healthcare professionals (that we aim to gather from the QUAL synthesis)	MM inferences providing an overall interpretation of what is associated with the ECHO Model success (or failure) in developing healthcare professionals’ competencies

#### First step: Descriptive synthesis

Prior to undertaking QUAN and QUAL syntheses, we will summarize all included QUAN, QUAL and MM studies regarding their characteristics, population, context and settings in a table format. Informal evidence extracted using the GREET checklist on ECHO programs’ components/characteristics will also be summarized in a table, including QUAN, QUAL and MM studies.

#### Second step: QUAN synthesis

##### Summary intervention effects and meta-analyses

To evaluate the effectiveness of the ECHO Model on the development of competencies in healthcare professionals, we will synthesize all intervention effect estimates for each outcome of interest using meta-analyses. Meta-analyses will be undertaken using the Cochrane Collaboration Review Manager RevMan version 5.4.1 (© 2020 The Cochrane Collaboration, London, UK; www.training.cochrane.org). All results will be expressed with 95% confidence intervals (CI). Statistically significant results will be defined with a two-sided alpha of 0.05.

A minimum of two studies will be needed to contribute to a meta-analysis. To minimize heterogeneity, we will favor the pooling of studies in which the comparators (active or passive comparison groups) and the outcomes of interest (objective or subjective outcome measures of competencies) are similar. Based on existing systematic reviews on the ECHO Model [9, 42], we currently expect that all outcomes of interest were mostly measured as continuous variables and using different instruments. As such, we will use an inverse variance approach for continuous outcomes and random effect models in all meta-analyses. All results will be expressed as standardized mean differences. We will interpret the significance of effect sizes using Cohen’s classification (< 0.2 = negligible; 0.2—0.49 = small; 0.5—0.8 = moderate; > 0.8 = large) [67].

In each meta-analysis, we will assess statistical heterogeneity, which is the inconsistency in intervention effect estimates between studies that is not due to chance, using the *X*^2^ test and the *I*^2^ statistic. A statistically significant *p* value at the *X*^2^ test or an *I*^2^ statistic > 50% will be considered as indicative of high statistical heterogeneity. Where statistical pooling is not possible the findings will be presented in narrative form including tables and figures.

##### Subgroup and sensitivity analyses

Since we anticipate heterogeneity between ECHO programs included in the QUAN component of the review [9], we plan to carry out subgroup analyses to investigate potential statistical heterogeneity sources when four or more studies are included in a single meta-analysis (two in each subgroup). If there are a sufficient number of studies, we will explore the following potential effect modifiers:

- Intervention: topic(s) or health condition(s) targeted in the program
- Population: professional group(s) participating in the program
- Context: practice setting of participating healthcare professionals
- Study design: QUAN randomized vs. QUAN non-randomized controlled studies

Based on the MMAT final interpretation of QUAN studies’ methodological quality, sensitivity analyses will also be conducted to exclude studies of low methodological quality (i.e., two or fewer criteria met out of five).

##### Assessment of reporting biases

Based on Cochrane recommendations [54], we will assess reporting biases using funnel plots if more than 10 studies are included in a single meta-analysis. We will follow the guidelines regarding funnel plot asymmetry as described in the Cochrane Handbook for Systematic Reviews of Interventions version 6.1 [54].

#### Third step: QUAL synthesis

Thirdly, a thematic synthesis [68] of the QUAL findings from the QUAL and MM studies (QUAL component only) will be undertaken by two independent reviewers using the MAXQDA Standard software version 2020.1. This will assist in understanding what influences the development of competencies in healthcare professionals participating in ECHO.

To ensure consistency and coherence between each reviewer’s coding, we will use a deductive approach to data reduction—i.e., organization of the mass of QUAL data and discarding of irrelevant data [69]. To achieve this, a set of three conceptual categories that depict the nature of potential influences on the development of competencies in healthcare professionals will serve as an initial path for organizing the QUAL raw data. In line with our conceptual representation of the ECHO Model [34], these three conceptual categories will comprise of educational factors (intervention components/characteristics), personal factors (e.g., demographics, motivation, engagement, ability to use technology, openness to change) and contextual factors (interpersonal, institutional, organizational, community, and sociopolitical influences). However, this stage of the review will remain iterative to enable new categories to be developed as needed, based on the similarity and recurrence of the data.

During this process, each study will be read and reread to enable the reviewer to familiarize themselves with the study results and the methods used. Then, two independent reviewers will scrutinize the study results for meaningful units with regards to the QUAL review question. Data will be coded line by line to assign the content of each line or sentence under one of the established conceptual categories. Any changes or differences arising from the coding system will be resolved with two reviewers and we will bring in a third reviewer in case of a persistent disagreement or uncertainty. Irrelevant information will be kept in an independent category to ensure that we have future access, as unexpected findings may call for re-examination of some data previously considered unnecessary.

In the following step, the lead review author (GC) will examine each code to identify recurrence or patterns in the data. Subsequently, a first set of themes and subthemes will be created, through assembling the data and displaying the data into the form of a hierarchy. These initial sets of themes and subthemes will then be synthesized by examining the themes within and across each study, based on meaning similarity. Themes will be refined and renamed with the review authors until the synthesized findings provide an answer to the QUAL review question. The QUAL findings will be presented in narrative form including each emergent theme with supporting quotes (i.e., example of results/themes drawn from the studies included in the QUAL component of the review).

#### Fourth step: Integration of QUAN and QUAL evidence

At the final stage, findings of each synthesis will be compared and contrasted to produce an overall configured synthesis and interpretation of what influences the ECHO Model effectiveness on the development of competencies in healthcare professionals. This will involve QUAN and QUAL evidence being simultaneously juxtaposed, for the purpose of interrogating how the experiences/views of ECHO’s participants can help explain the model effectiveness in developing healthcare professionals’ competencies. This juxtaposition will assist the review team in exploring the heterogeneity between the QUAN findings (intervention components/characteristics and effect size measures) and in interpreting, based on the QUAL findings (themes), under which influences some ECHO programs were effective—or more effective—and some were not. Integration of both sets of evidence will be attained by performing a comparison strategy [70], which will assist in considering where the QUAN and QUAL findings of the review agree (correspondence, similarities), offer complementary information, or are in contradiction (disagreement or dissonance).

The comparison integration strategy will be performed using a matrix approach [71, 72]. A matrix will allow us to closely map the findings of the review on a single table and conduct a side-by-side comparison to identify matches and mismatches [72]. We will organize the matrix in a *theme-by-effect size* configuration [73]. This matrix will help to identify contradictions and similarities between the QUAL and QUAN findings, aspects in QUAN and QUAL evidence that are not explored, as well as to explain why the intervention is effective or not, and why there are differences in direction and effect size between QUAN studies [46]. Where juxtaposition is not possible, the findings will be presented in narrative form. An example of the planned matrix inspired by Candy’s et al. [74] MMSR can be found in Additional file 4.

## Discussion

### Added value of the review

This protocol outlines the process to be undertaken for a MMSR aiming to gather evidence on the ECHO Model. To our knowledge, no review has yet selected, appraised and synthesized evidence from QUAN (and the QUAN component of MM studies) and QUAL (and the QUAL component of MM studies) studies for the overarching aim of comparison and complementarity between both strands of findings. As opposed to single method reviews, this MMSR has the potential to offer a more complete synthesis of the available evidence and therefore contribute to the current body of knowledge regarding the ECHO model.

This MMSR is necessary to explore in which conditions the model is most effective in developing healthcare professionals’ competencies. Furthermore, no review has focused on gathering and synthesizing evidence on how replications of ECHO-affiliated programs are implemented in various contexts, sometimes reflecting the particular topics and learning objectives addressed in a given program [45]. Hence, this MMSR protocol was developed to elucidate these variations in the numerous replications of the ECHO Model and provide a clear understanding of which components may lead to better outcomes in healthcare professionals and patients' health. Overall, this review will integrate evidence from diverse methodologies in a systematic way, which should help shed new light on the ECHO Model.

### Strengths and limitations

A key strength of this review is the proposed methodology, which is crucial and relevant to the MMSR methodological field. For instance, this review protocol was developed in accordance with the current recommendations in the literature on MMSR [46, 50, 71, 72, 75], entailing a rigorously and thoughtfully articulated convergent segregated design. Further, this protocol has emphasized on clear procedures, steps and tools, and has provided an explicit wording on when and how integration will be occurring throughout the review process. Thus, we believe that this protocol might offer appropriate and meaningful guidance in conducting and reporting MMSR, and perhaps contribute to methodology advancement.

However, this MMSR has some potential limitations. Regarding the search strategy, this review will be including empirical studies exclusively, which means that other sources of existing information will be excluded from the outset. While other forms of evidence might be an interesting addition to the state of knowledge in terms of comprehensiveness, this decision was made on the grounds that there is a considerable amount of eligible and primary studies. Indeed, based on a previous review that focused on a similar body of literature [9], 52 empirical studies from peer-reviewed articles were included by these authors. Also, only papers of English and French-language studies will be included in this review. To provide an overview of the available studies on the ECHO Model and a clear picture of the potential language bias, studies excluded on the basis of language will be identified and reported in full in the completed review.

Another potential limitation is that all studies meeting the eligibility criteria will be included in the review, meaning that no study will be excluded based on low methodological quality. Since risk of bias and lack of rigor are primary concerns when undertaking a MMSR [46], all included studies will be critically appraised using the latest version of the MMAT. To ensure transparency and enhance rigor, a table indicating the ratings for each criterion of all included studies will be developed using the MMAT and will be reported in full.

Although this review will be restricted to ECHO-affiliated programs only to limit clinical diversity, we anticipate that programs’ characteristics of included QUAN studies will vary in terms of population (targeted professional groups), topics (targeted health condition or disease), participants’ exposure to the intervention (frequency and duration of the program) and context of care delivery (e.g., community services, primary care and hospital). However, the richness of the QUAL findings that we expect to gather will assist in explaining any potential variation in the program effectiveness on the QUAN outcomes. Another downside of including studies of ECHO-affiliated programs exclusively is that the findings generated from this review may not be generalizable to other technology-enabled collaborative learning models involving healthcare professionals such as virtual communities of practice and networks. However, we believe that these models differ in terms of educational principles and learning methods, and thus do not meet the aim and scope of this review.

Finally, considering that this review builds on a MM approach, a potential challenge to consider is the complexity associated with the incorporation of evidence derived from a range of research designs into one single synthesis [75]. To address any practical issues during synthesis and integration, the process will follow the available guidance for MMSR [44] and the overall interpretation of the evidence will be reviewed independently by each team member. A full immersion of the lead author in the entirety of the evidence base, extended reflection with potential explanations in case of divergences between QUAN and QUAL findings and transparency in the reporting of the integration process will provide greater understanding of and insight into the evidence. Further, the composition of the review team was built with the objective of bringing together researchers with experience in a diversity of research field (i.e., ICTs, competency development, QUAN and QUAL research designs, MMSR), each of them adding complementary and relevant expertise for this MMSR. This will help in providing further guidance and in ensuring that both content and methodological aspects of the review are adequately addressed.

### Dissemination and implication of the review findings in practice

The dissemination plan includes standard and innovative (e.g., website portals, social media, Project ECHO Networks, knowledge exchange events with clinical administrators, healthcare professionals, key stakeholders) means of ensuring that the review findings will be communicated regionally, nationally and internationally, and accessible for a broad audience. This MMSR findings—including the QUAN, QUAL and MM findings—will be disseminated through publication in relevant peer-reviewed journals and presented at suitable fora including academic, scientific and professional conferences in the field of ICTs and CE in the health professions. The strengths, limitations and recommendations to improve the development, implementation and/or evaluation of ECHO-affiliated programs will be discussed in the completed review.

In conclusion, this MMSR approach will contribute to further our understanding of what influences the ECHO model effectiveness in developing healthcare professionals’ competencies. The evidence gathered from QUAN, QUAL and MM studies will be maximized to illustrate the best ways to implement the ECHO Model as an effective intervention, and will be useful in guiding future research and educational practices in this area. This is essential to assist in clinical, organizational and policy decision-making [44]. The review findings may be applied internationally and across all health disciplines. It is expected that the review findings will be valuable to researchers, academicians and other stakeholders (e.g., patients, policymakers, administrators, healthcare professionals, educators) regarding areas of improvements in future replications of the ECHO Model.

## Supplementary Information

**Additional file 1: **Examples of educational methods delivered in implementations of ECHO-affiliated programs. **Table 1.** The ECHO Model educational theories, principles and methods with concrete examples of delivery.

**Additional file 2.** Recommended items to address in a systematic review protocol from the Preferred Reporting Items for Systematic review and Meta-Analysis Protocols (PRISMA-P) 2015 checklist [47].

**Additional file 3.** Complete search strategy for the electronic databases.

**Additional file 4: **Example of a matrix for integrating the QUAN and QUAL findings of the review, inspired from Candy *et al.* (2011) [74]. **Table 3.** Tabulation of critical factors (1 - …) drawn from the QUAL findings (themes), categorized according to nature of factors [34], with each ECHO program components/characteristics and effect size (E) on healthcare professionals’ competency development.

## Data Availability

No data are yet available.

## Abbreviations

CE: Continuing Education
CI: Confidence Interval
CINAHL: Cumulative Index to Nursing & Allied Health Literature
ECHO: Extension for Community Healthcare Outcomes
GREET: Guideline for Reporting Evidence-based practice Educational interventions and Teaching
ICTs: Information and Communication Technologies
JBI: Joanna Briggs Institute
MM: Mixed Methods
MMAT: Mixed Methods Appraisal Tool
MMSR: Mixed Methods Systematic Review
PICO: Population—Intervention—Comparator—Outcomes
PICo: Population—phenomenon of Interest—Context
PRISMA-P: Preferred Reporting Items for Systematic review and Meta-Analysis Protocols
QUAL: Qualitative
QUAN: Quantitative
RCT: Randomized Controlled Trial
