# Supplementary material for: Effectiveness and experiences of the Extension for Community Healthcare Outcomes (ECHO) Model in developing competencies among healthcare professionals: a mixed methods systematic review protocol
Source: Syst Rev. 2021 Dec 16;10:313. doi: 10.1186/s13643-021-01832-0 (PMC8675457; doi:10.1186/s13643-021-01832-0)
Supplement: Supplementary file 1 — Additional file 1: Examples of educational methods delivered in implementations of ECHO-affiliated programs. Table 1. The ECHO Model educational theories, principles and methods with concrete examples of delivery. [file 13643_2021_1832_MOESM1_ESM.pdf]

Additional file 1: Examples of educational methods delivered in implementations of ECHO-affiliated programs

Table 1. The ECHO Model educational theories, principles and methods with concrete examples of delivery

Educational theories	Educational principles	Educational methods and examples of delivery
Social cognitive theory	Learners must believe that the benefits of performing the new behavior outweigh its costs, develop confidence in their ability to perform this behavior and receive reinforcement of positive behavior changes from persons who are seen as important	**Case-based discussion and reflexive practice:**<br>- Case-based presentation of patients from participants<br>- Discussion and questioning to stimulate reflexive practice<br>- Retroaction from specialists and peers<br>- Opportunity to try new behaviors in practice and receive feedback at a follow-up session<br>- Exposition to the benefits of peers' behavior changes
Situated learning theory	Effective learning goes through a novice-expert interaction, where novice learns to extend their current skills and knowledge by being motivated to perform an idealized and manageable version of a task	**Mentorship:**<br>- Program delivered through a Hub-and-spokes model including specialists and general healthcare professionals<br>- Realistic problems from practice to stimulate intrinsic interest<br>- Short didactic presentations tailored to learners' needs<br>- Evidence-based practice recommendations from specialists<br>- Focus given on knowledge that is useful and relevant to practice
Theory of communities of practice	Learning evolves through a process of continuous participation, engagement and sense of belonging in a collaborative community of learners with a common goal	**Group modality:**<br>- Emphasis on common basic values of the program (e.g., importance of introducing each participant, creating collaborative and respectful environment of learning)<br>- Sharing of knowledge and personal experiences<br>- Peer support and problem-solving in a collaborative effort
