# Supplementary material for: Effectiveness and experiences of the Extension for Community Healthcare Outcomes (ECHO) Model in developing competencies among healthcare professionals: a mixed methods systematic review protocol
Source: Syst Rev. 2021 Dec 16;10:313. doi: 10.1186/s13643-021-01832-0 (PMC8675457; doi:10.1186/s13643-021-01832-0)
Supplement: Supplementary file 2 — Additional file 2. Recommended items to address in a systematic review protocol from the Preferred Reporting Items for Systematic review and Meta-Analysis Protocols (PRISMA-P) 2015 checklist [47]. [file 13643_2021_1832_MOESM2_ESM.pdf]

**Additional file 2: Recommended items to address in a systematic review protocol from the Preferred Reporting Items for Systematic review and Meta-Analysis Protocols (PRISMA-P) 2015 checklist [48]**

| Section and topic | Item No | Checklist item | Manuscript reference |
|---|---|---|---|
| **ADMINISTRATIVE INFORMATION** | | | |
| Title: | | | |
| Identification | 1a | Identify the report as a protocol of a systematic review | Title Page; p.1 |
| Update | 1b | If the protocol is for an update of a previous systematic review, identify as such | NA |
| Registration | 2 | If registered, provide the name of the registry (such as PROSPERO) and registration number | Abstract section; p.5 |
| Authors: | | | |
| Contact | 3a | Provide name, institutional affiliation, e-mail address of all protocol authors; provide physical mailing address of corresponding author | Title Page; p.1-3 |
| Contributions | 3b | Describe contributions of protocol authors and identify the guarantor of the review | Main text; Authors' contributions section; p.31 |
| Amendments | 4 | If the protocol represents an amendment of a previously completed or published protocol, identify as such and list changes; otherwise, state plan for documenting important protocol amendments | NA |
| Support: | | | |
| Sources | 5a | Indicate sources of financial or other support for the review | Main text; Declarations section and Funding statement; p.31 |
| Sponsor | 5b | Provide name for the review funder and/or sponsor | NA |
| Role of sponsor or funder | 5c | Describe roles of funder(s), sponsor(s), and/or institution(s), if any, in developing the protocol | NA |
| **INTRODUCTION** | | | |
| Rationale | 6 | Describe the rationale for the review in the context of what is already known | Main text; Background Section (Current knowledge gaps about the ECHO Model and importance of the proposed systematic review subsection) p.10-12 |
| Objectives | 7 | Provide an explicit statement of the question(s) the review will address with reference to participants, interventions, comparators, and outcomes (PICO) | Main text; Background Section (Aim and review questions subsection); p.12 |
| **METHODS** | | | |

| | | | |
|---|---|---|---|
| Eligibility criteria | 8 | Specify the study characteristics (such as PICO, study design, setting, time frame) and report characteristics (such as years considered, language, publication status) to be used as criteria for eligibility for the review | Main text; Methods Section (Eligibility criteria subsection); p.13-18 |
| Information sources | 9 | Describe all intended information sources (such as electronic databases, contact with study authors, trial registers or other grey literature sources) with planned dates of coverage | Main text; Methods Section (Search strategy for identification of studies subsection); p.18-19 |
| Search strategy | 10 | Present draft of search strategy to be used for at least one electronic database, including planned limits, such that it could be repeated | Additional file 3 |
| Study records: | | | |
| Data management | 11a | Describe the mechanism(s) that will be used to manage records and data throughout the review | Main text; Methods Section (Selection of studies subsection); p.20 |
| Selection process | 11b | State the process that will be used for selecting studies (such as two independent reviewers) through each phase of the review (that is, screening, eligibility and inclusion in meta-analysis) | Main text; Methods Section (Selection of studies subsection); p.20 |
| Data collection process | 11c | Describe planned method of extracting data from reports (such as piloting forms, done independently, in duplicate), any processes for obtaining and confirming data from investigators | Main text; Methods Section (Data extraction and management subsection); p.21-23 |
| Data items | 12 | List and define all variables for which data will be sought (such as PICO items, funding sources), any pre-planned data assumptions and simplifications | Main text; Methods Section (Data extraction and management subsection); p.21-23 |
| Outcomes and prioritization | 13 | List and define all outcomes for which data will be sought, including prioritization of main and additional outcomes, with rationale | Main text; Methods Section (Outcomes subsection); p.15-16 |
| Risk of bias in individual studies | 14 | Describe anticipated methods for assessing risk of bias of individual studies, including whether this will be done at the outcome or study level, or both; state how this information will be used in data synthesis | Main text; Methods Section (Assessment of methodological quality subsection); p.20-21 |
| Data synthesis | 15a | Describe criteria under which study data will be quantitatively synthesised | Main text; Methods Section (Second step: Quantitative synthesis subsection); p.24-26 |
| | 15b | If data are appropriate for quantitative synthesis, describe planned summary measures, methods of handling data and methods of combining data from studies, including any planned exploration of consistency (such as $I^2$, Kendall's τ) | Main text; Methods Section (Second step: Quantitative synthesis subsection); p.24-26 |
| | 15c | Describe any proposed additional analyses (such as sensitivity or subgroup analyses, meta-regression) | Main text; Methods Section (Subgroup and sensitivity analysis subsection); p.24-25 |
| | 15d | If quantitative synthesis is not appropriate, describe the type of summary planned | Main text; Methods Section (First step: Descriptive |

| | | | |
|---|---|---|---|
| | | | synthesis subsection); p.24 |
| Meta-bias(es) | 16 | Specify any planned assessment of meta-bias(es) (such as publication bias across studies, selective reporting within studies) | Main text; Methods Section (Assessment of reporting biases subsection) findings; p.26 |
| Confidence in cumulative evidence | 17 | Describe how the strength of the body of evidence will be assessed (such as GRADE) | NA (assessment of confidence in cumulative evidence are currently not recommended in mixed methods systematic reviews) [45] |

*Note.* NA = Not applicable.
* This review protocol was drafted and appraised according to the PRISMA-P statement [48] together with the PRISMA-P 2015 Explanation and Elaboration paper [49] and the PRISMA 2020 updated guidance [50].
