# Supplementary material for: Effectiveness and experiences of the Extension for Community Healthcare Outcomes (ECHO) Model in developing competencies among healthcare professionals: a mixed methods systematic review protocol
Source: Syst Rev. 2021 Dec 16;10:313. doi: 10.1186/s13643-021-01832-0 (PMC8675457; doi:10.1186/s13643-021-01832-0)
Supplement: Supplementary file 3 — Additional file 3. Complete search strategy for the electronic databases. [file 13643_2021_1832_MOESM3_ESM.pdf]

Additional file 3: Complete search strategy for the electronic databases

MEDLINE(R) ALL 1946 to October 01, 2020 [OVID]

1	Extension for Community Healthcare Outcomes.tw,sh,kf.	113
2	((project* or program* or telementor* or tele-mentor* or model* or videoconferen*) adj5 (ECHO or ECHO's or virtual consultation*)).tw,sh,kf.	745
3	(SCAN-ECHO or tele-ECHO).tw,sh,kf.	43
4	((project* or mentor* or telementor* or tele-mentor* or videoconferen*) adj5 ECHO*).tw,sh,kf.	411
5	(legitimate peripheral participation* or "hub-and-spoke").tw,sh,kf.	435
6	((communit* or "situated learning" or network* or knowledge or VCoPs) adj5 (ECHO or ECHO's)).tw,sh,kf.	371
**7**	**or/1-6**	**1620**
8	(virtual communit* of practice or "VCoPs" or "knowledge network" or "situated learning" or "virtual community of practice" or knowledge communit* or "virtual collaboration" or network* of practice or virtual knowledge-sharing communit* or virtual communit* of learning or virtual collaborative communit* or online learning communit* or virtual learning communit*).tw,sh,kf.	724
9	((virtual or online or distance or web or internet or remote or Facebook or social network* or Twitter or Instagram or Linkedin or doximity or technolog*) adj2 (practic* or learning or collaboration or knowledge or mentorship or educat*) adj2 (communit* or network* or group or Forum* or blog* or exchang*)).tw,sh,kf.	836
**10**	**8 or 9**	**1504**
11	(tele-mentor* or telementor* or telemedicine or telehealth).tw,kf.	18406
12	Education, Continuing/ or Online Systems/ or telemedicine/	41154
13	exp Videoconferencing/	1954
14	(videoconferenc* or video-conferenc* or tele-educat* or tele-conferenc* or teleconferenc* or teletrain* or tele-train* or teleconsult* or tele-consult* or teletrain* or online learn* or online mentor* or virtual consult* or virtual communit* of practice or VCoPs or virtual community of learning or virtual collaboration* or knowledge network* or distance learn* or collaborative learn* or distance education* or online technolog*).tw,kf.	10706
15	Education, Medical, Continuing/ or Education, Distance/	28686
16	Remote Consultation/ or Distance Counseling/	4909
17	Computer-Assisted Instruction/	11900
**18**	**or/11-17**	**95668**
19	Tertiary Care Centers/	14126
20	Academic Medical Centers/	18967
21	Hospitals, University/	30014

22	Hospitals, Urban/	7407
23	Specialization/ or specialism/	24414
24	(speciali* or academic medical cent* or university hospital* or tertiary care cent* or specialty or academic health cent*).ti,kw.	55102
**25**	**or/19-24**	**130644**
26	Physicians/ or Physicians, Primary Care/ or Physicians, Family/	109206
27	General Practitioners/	8009
28	Nurses/	39274
29	Nurse Practitioners/ or Family Nurse Practitioners/	17740
30	(physician* or nurse* or nursing).ti,kw.	374609
31	(general practitioner* or generalist*).ti,kw.	16724
32	Rural Health/ or Hospitals, Rural/ or Rural Health Services/	39904
33	Primary Health Care/	78241
34	Medically Underserved Area/	7036
35	Community Health Services/	31756
36	Rural Nursing/	109
37	(rural adj2 health).tw,sh.	7190
38	(rural adj2 hospital*).tw,sh.	5732
39	(medically underserved area* or remote area*).tw,sh.	11955
40	rural nurs*.tw,sh.	628
**41**	**or/26-40**	**616388**
**42**	**18 and 25 and 41**	**764**
**43**	**7 or 10 or 42**	**3850**
**44**	**limit 43 to yr="2003 -Current"**	**3040**

Embase 1980 to 2020 Week 40 [OVID]

1	Extension for Community Healthcare Outcomes.tw,kw.	192
2	((project* or program* or telementor* or tele-mentor* or model* or videoconferen*) adj2 (ECHO or ECHO's or virtual consultation*)).tw,kw.	574
3	(SCAN-ECHO or tele-ECHO).tw,kw.	67
4	((project* or mentor* or telementor* or tele-mentor* or videoconferen*) adj2 ECHO*).tw,kw.	375
5	(legitimate peripheral participation* or "hub-and-spoke").ti,kw.	187
6	((communit* or "situated learning" or network* or knowledge or VCoPs) adj2 (ECHO or ECHO's)).tw,kw.	252
7	or/1-6	1142

8	(virtual communit* of practice or "VCoPs" or "knowledge network" or "situated learning" or "virtual community of practice" or knowledge communit* or "virtual collaboration" or network* of practice or virtual knowledge-sharing communit* or virtual communit* of learning or virtual collaborative communit* or online learning communit* or virtual learning communit*).tw,kw.	956
9	((virtual or online or distance or web or internet or remote or Facebook or social network* or Twitter or Instagram or Linkedin or doximity or technolog*) adj2 (practic* or learning or collaboration or knowledge or mentorship or educat*) adj2 (communit* or network* or group or Forum* or blog* or exchang*)).tw,kw.	744
10	8 or 9	1630
11	(tele-mentor* or telementor* or telemedicine or telehealth).tw,kw.	24048
12	continuing education/ or online system/ or telemedicine/	81969
13	videoconferencing/	4386
14	(videoconferenc* or video-conferenc* or tele-educat* or tele-conferenc* or teleconferenc* or teletrain* or tele-train* or teleconsult* or tele-consult* or teletrain* or online learn* or online mentor* or virtual consult* or virtual communit* of practice or VCoPs or virtual community of learning or virtual collaboration* or knowledge network* or distance learn* or collaborative learn* or distance education* or online technolog*).tw,kw.	14472
15	teleconsultation/	10095
16	e-counseling/	233
17	or/11-16	108975
18	tertiary care center/	62054
19	university hospital/	125395
20	urban hospital/	500
21	specialization/	6902
22	(speciali* or academic medical cent* or university hospital* or tertiary care cent* or specialty or academic health cent*).ti,kw.	71992
23	or/18-22	242535
24	physician/	281764
25	general practitioner/	97132
26	nurse/	122085
27	nurse practitioner/	23470
28	family nurse practitioner/	134
29	(physician* or nurse* or nursing).ti,kw.	352371
30	(general practitioner* or generalist*).ti,kw.	18189
31	Rural Health/ or Hospitals, Rural/ or Rural Health Services/	13565

32	primary health care/	64655
33	community care/	52458
34	rural health nursing/	163
35	(rural adj2 health).tw.	7846
36	(rural adj2 hospital*).tw.	7111
37	(medically underserved area* or remote area*).tw.	6860
38	rural nurs*.tw.	551
39	or/24-38	862441
40	17 and 23 and 39	864
41	7 or 10 or 40	3582

All EBM Reviews - Cochrane DSR, ACP Journal Club, DARE, CCA, CCTR, CMR, HTA, and NHSEED [OVID]

1	Extension for Community Healthcare Outcomes.af.	6
2	((project* or program* or telementor* or tele-mentor* or model* or videoconferen*) adj2 (ECHO or ECHO's or virtual consultation*)).af.	31
3	(SCAN-ECHO or tele-ECHO).af.	8
4	((project* or mentor* or telementor* or tele-mentor* or videoconferen*) adj2 ECHO*).af.	18
5	(legitimate peripheral participation* or "hub-and-spoke").ti,kw.	9
6	((communit* or "situated learning" or network* or knowledge or VCoPs) adj2 (ECHO or ECHO's)).af.	9
7	or/1-6	62
8	(virtual communit* of practice or "VCoPs" or "knowledge network" or "situated learning" or "virtual community of practice" or knowledge communit* or "virtual collaboration" or network* of practice or virtual knowledge-sharing communit* or virtual communit* of learning or virtual collaborative communit* or online learning communit* or virtual learning communit*).af.	89
9	((virtual or online or distance or web or internet or remote or Facebook or social network* or Twitter or Instagram or Linkedin or doximity or technolog*) adj2 (practic* or learning or collaboration or knowledge or mentorship or educat*) adj2 (communit* or network* or group or Forum* or blog* or exchang*)).af.	228
10	8 or 9	309
11	(tele-mentor* or telementor* or telemedicine or telehealth).af.	5763
12	continuing education/ or online system/ or telemedicine/	2557
13	videoconferencing/	190

14	(videoconferenc* or video-conferenc* or tele-educat* or tele-conferenc* or teleconferenc* or teletrain* or tele-train* or teleconsult* or tele-consult* or teletrain* or online learn* or online mentor* or virtual consult* or virtual communit* of practice or VCoPs or virtual community of learning or virtual collaboration* or knowledge network* or distance learn* or collaborative learn* or distance education* or online technolog*).af.	2559
15	teleconsultation/	441
16	e-counseling/	0
17	or/11-16	8135
18	tertiary care center/	2
19	university hospital/	1007
20	urban hospital/	187
21	specialization/	113
22	(speciali* or academic medical cent* or university hospital* or tertiary care cent* or specialty or academic health cent*).ti,kw.	10027
23	or/18-22	11167
24	physician/	896
25	general practitioner/	31
26	nurse/	1
27	nurse practitioner/	337
28	family nurse practitioner/	2
29	(physician* or nurse* or nursing).ti,kw.	32194
30	(general practitioner* or generalist*).ti,kw.	3405
31	Rural Health/ or Hospitals, Rural/ or Rural Health Services/	963
32	primary health care/	4661
33	community care/	14
34	rural health nursing/	0
35	(rural adj2 health).af.	1855
36	(rural adj2 hospital*).af.	423
37	(medically underserved area* or remote area*).af.	518
38	rural nurs*.tw.	10
39	or/25-38	41820
40	17 and 24 and 40	87
41	7 or 10 or 40	453

CINAHL COMPLETE [EBSCO]

#	Question	Résultats

S1	TI Extension for Community Healthcare Outcomes OR TI (((project* or program* or telementor* or tele-mentor* or model* or videoconferen*) N2 (ECHO or ECHO's or virtual consultation*))) OR TI ((SCAN-ECHO or tele-ECHO)) OR TI (((project* or mentor* or telementor* or tele-mentor* or videoconferen*) N1 ECHO*)) OR TI ((legitimate peripheral participation* or "hub-and-spoke")) OR TI (((communit* or "situated learning" or network* or knowledge or VCoPs) N2 (ECHO or ECHO's)))	174
S2	AB Extension for Community Healthcare Outcomes OR AB (((project* or program* or telementor* or tele-mentor* or model* or videoconferen*) N2 (ECHO or ECHO's or virtual consultation*))) OR AB ((SCAN-ECHO or tele-ECHO)) OR AB (((project* or mentor* or telementor* or tele-mentor* or videoconferen*) N1 ECHO*)) OR AB ((legitimate peripheral participation* or "hub-and-spoke")) OR AB (((communit* or "situated learning" or network* or knowledge or VCoPs) N2 (ECHO or ECHO's)))	351
S3	TI (virtual communit* of practice or "VCoPs" or "knowledge network" or "situated learning" or "virtual community of practice" or knowledge communit* or "virtual collaboration" or network* of practice or virtual knowledge-sharing communit* or virtual communit* of learning or virtual collaborative communit* or online learning communit* or virtual learning communit*)) OR AB (virtual communit* of practice or "VCoPs" or "knowledge network" or "situated learning" or "virtual community of practice" or knowledge communit* or "virtual collaboration" or network* of practice or virtual knowledge-sharing communit* or virtual communit* of learning or virtual collaborative communit* or online learning communit* or virtual learning communit*))	522
S4	TI (((virtual or online or distance or web or internet or remote or Facebook or social network* or Twitter or Instagram or Linkedin or doximity or technolog*) N2 (practic* or learning or collaboration or knowledge or mentorship or educat*) N2 (communit* or network* or group or Forum* or blog* or exchang*))) OR AB (((virtual or online or distance or web or internet or remote or Facebook or social network* or Twitter or Instagram or Linkedin or doximity or technolog*) N2 (practic* or learning or collaboration or knowledge or mentorship or educat*) N2 (communit* or network* or group or Forum* or blog* or exchang*)))	953
S5	S1 OR S2	431
S6	S3 OR S4	1,356
S7	TI ((tele-mentor* or telementor* or telemedicine or telehealth)) OR AB ((tele-mentor* or telementor* or telemedicine or telehealth)) OR TI ((videoconferenc* or video-conferenc* or tele-educat* or tele-conferenc* or teleconferenc* or teletrain* or tele-train* or teleconsult* or tele-consult* or teletrain* or online learn* or online mentor* or virtual consult* or virtual communit* of practice or VCoPs or virtual community of learning or virtual collaboration* or knowledge network* or distance learn* or collaborative learn* or distance education* or online technolog*)) OR AB ((videoconferenc* or video-conferenc* or tele-educat* or tele-conferenc* or teleconferenc* or teletrain* or tele-train* or teleconsult* or tele-consult* or teletrain* or online learn* or online mentor* or virtual consult* or virtual communit* of practice or VCoPs or virtual community of learning or virtual collaboration* or knowledge network* or distance learn* or collaborative learn* or distance education* or online technolog*))	15,590
S8	(MH "Education, Continuing") OR (MH "Education, Medical, Continuing") OR (MH "Online Systems") OR (MH "Telemedicine") OR (MH "Videoconferencing") OR (MH "Telenursing") OR (MH "Telehealth") OR (MH "Remote Consultation")	47,107
S9	S7 OR S8	54,421
S10	((MH "Tertiary Health Care") OR (MH "Academic Medical Centers") OR (MH "Specialization")) OR TI ((speciali* or academic medical cent* or university hospital* or tertiary care cent* or specialty or academic health cent*)) OR AB ((speciali* or academic medical cent* or university hospital* or tertiary care cent* or specialty or academic health cent*))	200,326
S11	(MM "Physicians") OR (MH "Physicians, Family") OR (MH "Nurses") OR (MH "Nurse Practitioners") OR (MH "Family Nurse Practitioners") OR (MH "Rural Health") OR (MH "Rural Health Services") OR (MH "Primary Health Care") OR (MH "Hospitals, Rural") OR (MH "Rural Health Centers") OR (MH "Rural Health Nursing")	208,039

S12	TI ((physician* or nurse* or nursing)) OR TI ((general practitioner* or generalist*)) OR TI ((rural) N2 (health or hospital)) OR AB ((rural) N2 (health or hospital)) OR TI ((medically underserved area* or remote area*)) OR AB ((medically underserved area* or remote area*)) OR TI rural nurs* OR AB rural nurs*	413,241
S13	S11 OR S12	553,817
S14	S9 AND S10 AND S13	1,085
S15	S5 OR S6 OR S14	2,811
S16	Date de publication: 20030101-20211231	2,581

APA PsycInfo 1987 to October Week 3 2020 [OVID]

#	Searches	Results
1	Extension for Community Healthcare Outcomes.tw.	14
2	(SCAN-ECHO or tele-ECHO).tw.	6
3	((project* or program* or telementor* or tele-mentor* or model* or videoconferen*) adj5 (ECHO or ECHO's or virtual consultation*)).tw.	120
4	((project* or mentor* or telementor* or tele-mentor* or videoconferen*) adj5 ECHO*).tw.	47
5	(legitimate peripheral participation* or "hub-and-spoke").tw.	238
6	((communit* or "situated learning" or network* or knowledge or VCoPs) adj5 (ECHO or ECHO's)).tw.	144
7	or/1-6	479
8	(virtual communit* of practice or "VCoPs" or "knowledge network" or "situated learning" or "virtual community of practice" or knowledge communit* or "virtual collaboration" or network* of practice or virtual knowledge-sharing communit* or virtual communit* of learning or virtual collaborative communit* or online learning communit* or virtual learning communit*).ti.	388
9	((virtual or online or distance or web or internet or remote or Facebook or social network* or Twitter or Instagram or Linkedin or doximity or technolog*) adj2 (practic* or learning or collaboration or knowledge or mentorship or educat*) adj2 (communit* or network* or group or Forum* or blog* or exchang*)).ti.	365
10	*Online Community/	733
11	8 or 9 or 10	1338
12	(tele-mentor* or telementor* or telemedicine or telehealth).tw.	3612
13	(videoconferenc* or video-conferenc* or tele-educat* or tele-conferenc* or teleconferenc* or teletrain* or tele-train* or teleconsult* or tele-consult* or teletrain* or online learn* or online mentor* or virtual consult* or virtual communit* of practice or VCoPs or virtual community of learning or virtual collaboration* or knowledge network* or distance learn* or collaborative learn* or distance education* or online technolog*).tw.	13922
14	Continuing Education/	1545

15	Telemedicine/	5398
16	exp Videoconferencing/	599
17	Distance Education/	5497
18	Computer Assisted Instruction/	15055
19	or/12-18	34539
20	(speciali* or academic medical cent* or university hospital* or tertiary care cent* or specialty or academic health cent*).ti,sh.	6861
21	Physicians/ or Family Physicians/	20164
22	General Practitioners/	5644
23	Nurses/	25020
24	(physician* or nurse* or nursing).ti.	46413
25	(general practitioner* or generalist*).ti.	1798
26	Rural Health/	595
27	Primary Health Care/	18480
28	(rural adj2 health).ti.	493
29	(rural adj2 hospital*).ti.	146
30	(medically underserved area* or remote area*).ti.	84
31	rural nurs*.ti.	94
32	or/21-31	85497
33	19 and 20 and 32	29
34	7 or 11 or 33	1840
35	limit 34 to yr="2003 -Current"	1750
