# Supplementary material for: Effectiveness and experiences of the Extension for Community Healthcare Outcomes (ECHO) Model in developing competencies among healthcare professionals: a mixed methods systematic review protocol
Source: Syst Rev. 2021 Dec 16;10:313. doi: 10.1186/s13643-021-01832-0 (PMC8675457; doi:10.1186/s13643-021-01832-0)
Supplement: Supplementary file 4 — Additional file 4: Example of a matrix for integrating the QUAN and QUAL findings of the review, inspired from Candy et al. (2011) [74]. Table 3. Tabulation of critical factors (1 - …) drawn from the QUAL findings (themes), categorized according to nature of factors [34], with each ECHO program components/characteristics and effect size (E) on healthcare professionals’ competency development. [file 13643_2021_1832_MOESM4_ESM.pdf]

Additional file 4: Example of a matrix for integrating the QUAN and QUAL findings of the review, inspired from Candy *et al.* (2011) [75]

Table 3. Tabulation of critical factors (1 - …) drawn from the QUAL findings (themes), categorized according to nature of factors [34], with each ECHO program components/characteristics and effect size (E) on healthcare professionals' competency development

	QUAL component: Themes from the experiences/views of ECHO's participants							
	**Educational factors** (1 - 3)			**Personal factors** (4, 5)		**Contextual factors** (6 - …)		
**QUAN component: ECHO programs' components/ characteristics with effect size**	1	2	3	4	5	6	**…**	**E**
Reference of study A:<br>- Duration: 9 months<br>- Frequency: 2 hours weekly<br>- Topic: mental health and substance use disorders<br>- Population: primary care providers<br>- Educators: interdisciplinary panel of experts in co-occurring disorders<br>…	X	∅	—	X	X	∅	…	Effect size measure
Reference of study B:								
Reference of study C:								
Reference of study …								

Note. ECHO = Extension for Healthcare Community Outcomes; QUAL = Qualitative; QUAN = Quantitative. Legend. E = effect size; X = Commonality (correspondence) between an important factor and an ECHO program components/characteristics; ∅ = ECHO program components/characteristics do not correspond with an important factor; — = Insufficient information in a QUAN study to state whether an important factor was present in an ECHO program or not.
